# Supplementary material for: Cocaine-induced neuron subtype mitochondrial dynamics through Egr3 transcriptional regulation
Source: Mol Brain. 2021 Jun 29;14:101. doi: 10.1186/s13041-021-00800-y (PMC8240292; doi:10.1186/s13041-021-00800-y)
Supplement: Supplementary file 1 — Additional file 1. Human demographics for examined cocaine dependent and non-dependent tissue. [file 13041_2021_800_MOESM1_ESM.pdf]

SN #	Cocaine Dependence	Brain ID	Cause of Death	Age	Gender	Weight (g)	PH	Refrigeration Delay (hrs)	Comorbid Disorder
1	Cocaine dependent	7	Accident	51	Male	1428	6.73	1.5	Major Depression
2	Cocaine dependent	22	Natural	39	Male	1140	6.86	27.5	Depressive NOS
3	Cocaine dependent	26	Suicide	36	Male	1548	6.54	12	BiPolar Disorder
4	Cocaine dependent	33	Suicide	45	Male	1430	6.57	2.75	Major Depression
5	Cocaine dependent	34	Suicide	35	Male	1425	6.81	3.5	
6	Cocaine dependent	112	Suicide	24	Male	1460	6.89	2.5	
7	Cocaine dependent	120	Suicide	48	Male	1460	6.56	1.75	Major Depression
8	Cocaine dependent	121	Suicide	33	Male	1580	6.75	9	
9	Cocaine dependent	140	Suicide	43	Male	1445	6.78	5.25	Psychotic Disorder NOS
10	Cocaine dependent	147	Accident	24	Male	1480	6.33	7	
11	Cocaine dependent	156	Suicide	39	Male	1552	6.7	4.25	Major Depression
12	Cocaine dependent	193	Suicide	38	Male	1511.4	6.5	16	Depressive NOS
13	Cocaine dependent	194	Suicide	53	Male	1366.1	6.5	28	
14	Cocaine dependent	176	Suicide	50	Female	1255.5	6.3	7	
15	Not Cocaine Dependent	128	Suicide	46	Male	1600	6.83	12	
16	Not Cocaine Dependent	15	Natural	30	Male	1517	6.37	11	
17	Not Cocaine Dependent	17	Natural	41	Male	1376	6	3	
18	Not Cocaine Dependent	14	Natural	47	Male	1412	6.49	3.5	
19	Not Cocaine Dependent	20	Accident	32	Male	1516	6.67	4	
20	Not Cocaine Dependent	36	Natural	27	Male	1595	6.55	3	
21	Not Cocaine Dependent	94	Accident	15	Male	1420	6.72	16.75	
22	Not Cocaine Dependent	133	Accident	42	Male	1470	6.75	2.5	
23	Not Cocaine Dependent	135	Natural	18	Male	1470	6.87	2	
24	Not Cocaine Dependent	16	Accident	28	Male	1565	6.32	2.25	
25	Not Cocaine Dependent	197	Suicide	41	Female	1355.2	6.5	3.5	
26	Not Cocaine Dependent	173	Accident	20	Male	1533	6.3	12	
