# Supplementary material for: Cocaine-induced neuron subtype mitochondrial dynamics through Egr3 transcriptional regulation
Source: Mol Brain. 2021 Jun 29;14:101. doi: 10.1186/s13041-021-00800-y (PMC8240292; doi:10.1186/s13041-021-00800-y)
Supplement: Supplementary file 2 — Additional file 2. Primers utilized in study. [file 13041_2021_800_MOESM2_ESM.pdf]

	Forward	Reverse
mDrp1	GGGCACTTAAATTGGGCTCC	TGTATTCTGTTGGCGTGGAAC
mNrf2	TCTACTGAAAAGGCGGCTCA	TTGCCATCTCTGGTTTGCTG
mNRF1	AGACCTCTGCTAGATTCACCG	CCTGGACTTCACAAGCACTC
mPolG	ACTCCTGGAACAGTTGTGCT	CGTCCATCTACTCAGGACGG
mTFAMSV1	GCAGGCACTACAGCGATACA	GCCTCTACCTTTCCCATTCC
mTFB1	TACGCCCTTGATAGAGCCCA	TCCTTCGAAACTGAAACGCA
mTom20	CTGTGCTCTGGGCACTTAAC	AGGGTGCACACAGGTCTAAT
mGapdh	AGGTCGGTGTGAACGGATTTG	TGTAGACCATGTAGTTGAGGT CA
rDrp1	CTCCACCTTTTGAAGCCAGG	GCAGCCGTAGTCCTCAAAGA
rNrf2	CCGTACAAAACAAACACTAG CTC	AGATGGCAACGTGTTCCTTG
rNRF1	ATGGCGGAAGTAATGAAAGA CG	TACTTCCCAGCAGCCTTAGC
rPolG	CTCCGCACCCGAAGATTTG	CCTCCTCAGAGAATGGGCAG
rTFAMSV1	ATCAAGACTGTGCGTGCATC	AGAACTTCACAAACCCGCAC
rTFB1	TGAAGACCCACAACTCTTTGC	CAGCAGTTAGAACCCACAGC
rTom20	TGGAATGAGCCAGACACCAA	CACAGTTTGCCCTTATCCCC
rGapdh	TGGCCTCCAAGGAGTAAGAA	TGTGAGGGAGATGCTCAGTG
hDrp1	TCTTGGAGGACTATGGCAGC	CAAAGCAGTTTGCCTGTGGA
hNrf2	AGCATTGGAGTGTCAGTATGT T	ACTAGCCCAAATGGTGTCCA
hNRF1	GGTGCGCTGTGGAAACAATA	CAGTAGCTCAACGCATGACC
hPolG	TCACCAAAGGCTCCTTGGAA	CACGGGAGCAAATACAGAGC
hTFAMSV1	GGCACAGGAAACCAGTTAGG	ATGCTGGCAGAAGTCCATGA
hTFB1	TGCACTACGTGGAGCTTCTT	GTCACATCTGGTCATTGGCA
hTom20	TAGCCTTGTGAGCTTCGCTA	CAGCAGACGCATTCTCTCAC
rGapdh	TGTTCGTCATGGGTGTGAAC	GCAGGGATGATGTTCTGGAG
