# Supplementary material for: Cocaine-induced neuron subtype mitochondrial dynamics through Egr3 transcriptional regulation
Source: Mol Brain. 2021 Jun 29;14:101. doi: 10.1186/s13041-021-00800-y (PMC8240292; doi:10.1186/s13041-021-00800-y)
Supplement: Supplementary file 3 — Additional file 3. Statistical table. [file 13041_2021_800_MOESM3_ESM.pdf]

**Figure**	**Test**	**Test Statistic**	**N Per Group and Effect Sizes**
1a Drp1	Unpaired T-test	$t_{(10)}$=2.3,P=0.0448	6 Saline, 6 Cocaine; Cohens d=1.3238
1a Nrf1	Unpaired T-test	$t_{(12)}$=1.01,P=0.3335	7 Saline, 7 Cocaine; Cohens d=0.538
1a Nrf2	Unpaired T-test	$t_{(10)}$=2.27,P=0.0467	5 Saline, 7 Cocaine; Cohens d=1.001
1a Polγ	Unpaired T-test	$t_{(11)}$=2.33,P=0.0406	6 Saline, 7 Cocaine; Cohens d=0.992
1a Tfam	Unpaired T-test	$t_{(3)}$=1.13,P=0.3423	3 Saline, 4 Cocaine; Cohens d=1.162
1a Tfb1	Unpaired T-test	$t_{(4)}$=0.83,P=0.4548	3 Saline, 3 Cocaine; Cohens d=0.675
1a Tomm20	Unpaired T-test	$t_{(10)}$=1.7,P=0.1214	5 Saline, 7 Cocaine; Cohens d=0.830
2a Nrf1	Unpaired T-test	$t_{(11)}$=3.37,P=0.0064	6 Saline, 7 Cocaine; Cohens d=1.940
2a Nrf2	Unpaired T-test	$t_{(10)}$=4.67,P=0.0009	6 Saline, 6 Cocaine Cohens d=2.6963
2a Polγ	Unpaired T-test	$t_{(10)}$=1.15,P=0.2797	6 Saline, 6 Cocaine; Cohens d=0.660
2a Tfam	Unpaired T-test	$t_{(11)}$=1.83,P=0.0952	6 Saline, 7 Cocaine; Cohens d=1.052
2a Tfb1	Unpaired T-test	$t_{(10)}$=1.6,P=0.1414	6 Saline, 6 Cocaine; Cohens d=0.922
2a Tomm20	Unpaired T-test	$t_{(10)}$=2.76,P=0.0203	5 Saline, 7 Cocaine; Cohens d=1.705
2b Nrf1	Unpaired T-test	$t_{(9)}$=2.71,P=0.0244	5 Saline, 6 Cocaine; Cohens d=1.606
2b Nrf2	Unpaired T-test	$t_{(9)}$=1.92,P=0.0884	5 Saline, 6 Cocaine; Cohens d=1.168
2b Pgc1α	Unpaired T-test	$t_{(9)}$=1.93,P=0.0866	5 Saline, 6 Cocaine; Cohens d=1.202
2b Polγ	Unpaired T-test	$t_{(9)}$=1.86,P=0.0961	5 Saline, 6 Cocaine; Cohens d=1.106
2b Tfam	Unpaired T-test	$t_{(9)}$=0.66,P=0.5292	5 Saline, 6 Cocaine; Cohens d=0.383
2b Tfb1	Unpaired T-test	$t_{(9)}$=2.44,P=0.0379	5 Saline, 6 Cocaine; Cohens d=1.441
2b Tomm20	Unpaired T-test	$t_{(9)}$=2.49,P=0.0348	5 Saline, 6 Cocaine; Cohens d=1.475
2c Tfb1	Unpaired T-test	$t_{(27)}$=1.45,P=0.1599	14 Control, 15 Cocaine dependents; Cohens d=0.528
2c Nrf1	Unpaired T-test	$t_{(28)}$=1.43,P=0.1649	15 Control, 15 Cocaine dependents; Cohens d=0.521
2c Pgc1α	Unpaired T-test	$t_{(28)}$=2.05,P=0.0505	15 Control, 15 Cocaine dependents; Cohens d=0.746
2c Nrf2	Unpaired T-test	$t_{(23)}$=2.33,P=0.0296	12 Control, 13 Cocaine dependents; Cohens d=0.937
2c Polγ	Unpaired T-test	$t_{(26)}$=2.12,P=0.044	13 Control, 15 Cocaine dependents; Cohens d=0.779
2c Tfam	Unpaired T-test	$t_{(29)}$=0.29,P=0.7785	16 Control, 15 Cocaine dependents; Cohens d=0.1024
2c Tomm20	Unpaired T-test	$t_{(28)}$=1.27,P=0.2149	15 Control, 15 Cocaine dependents; Cohens d=0.463
3b Nrf1	Unpaired T-test	$t_{(8)}$=1.14,P=0.2883	4 Saline, 6 Cocaine; Cohens d=0.810
3b Nrf2	Unpaired T-test	$t_{(9)}$=2.05,P=0.071	5 Saline, 6 Cocaine; Cohens d=1.272
3b Polg	Unpaired T-test	$t_{(7)}$=0.66,P=0.5358	4 Saline, 5 Cocaine; Cohens d=0.448
3b TFAM	Unpaired T-test	$t_{(6)}$=3.38,P=0.015	4 Saline, 4 Cocaine; Cohens d=2.388
3b TFB1	Unpaired T-test	$t_{(10)}$=1.61,P=0.1402	6 Saline, 6 Cocaine; Cohens d=0.925
3b Tomm20	Unpaired T-test	$t_{(9)}$=2.24,P=0.0526	6 Saline, 5 Cocaine; Cohens d=1.318
3c Nrf1	Unpaired T-test	$t_{(9)}$=1.95,P=0.0836	5 Saline, 6 Cocaine; Cohens d=1.182
3c Nrf2	Unpaired T-test	$t_{(9)}$=2.01,P=0.0762	5 Saline, 6 Cocaine; Cohens d=1.181

3c Polg	Unpaired T-test	$t_{(9)}$=2.47,P=0.0362	5 Saline, 6 Cocaine; Cohens d=1.523
3c TFAM	Unpaired T-test	$t_{(9)}$=3.34,P=0.0088	5 Saline, 6 Cocaine; Cohens d=2.050
3c TFB1	Unpaired T-test	$t_{(9)}$=2.32,P=0.0459	5 Saline, 6 Cocaine; Cohens d=1.431
3c Tomm20	Unpaired T-test	$t_{(8)}$=5.41,P=0.0007	5 Saline, 5 Cocaine; Cohens d=3.421
4b Pgc1α	Two-way ANOVA	Drug: $F_{(1,18)}$=4.869, P=0.0406, Virus: $F_{(1,18)}$=4.257, P=0.0538, Interaction: $F_{(1,18)}$ = 9.398, p = 0.0067.	5 SS-miR Saline, 6 SS-miR Cocaine, 6 Egr3-miR Saline, 6 Egr3-miR Cocaine; Cohens d=0.772
4b Drp1	Two-way ANOVA	Drug: $F_{(1,20)}$=4.511, P=0.0463, Virus: $F_{(1,20)}$=12.67, P=0.0020, Interaction: $F_{(1,20)}$=12.67, p=0.0020,	5 SSmiR-Saline, SSmiR-Cocaine, 6 Egr3miR-Saline, 7 Egr3miR; Cohens d=1.214
4d frequency	Two-way ANOVA	Drug: $F_{(5,70)}$=73.19, P<0.0001, Virus: $F_{(3,14)}$=0.6858, P=0.5754, Interaction: $F_{(15, 70)}$=3.035, P=0.0009	4 SS-miR Saline , 4 SS-miR Cocaine 5 Egr3-miR Saline, 5 Egr3-miR Cocaine,
4d density	Two-way ANOVA	Drug: $F_{(1,14)}$= 3.441, P=0.0848, Virus: $F_{(1,14)}$=2.207, P=0.1595, Interaction: $F_{(1,14)}$=0.2823, P=0.6035	4 SS-miR Saline , 4 SS-miR Cocaine 5 Egr3-miR Saline, 5 Egr3-miR Cocaine,
4d index	Two-way ANOVA	Drug $F_{(1,14)}$=0.01078, P = 0.9188, Virus $F_{(1,14)}$=0.3075, P=0.5880, Interaction: $F_{(1,14)}$=3.919, P= 0.0678.	4 SS-miR Saline , 4 SS-miR Cocaine 5 Egr3-miR Saline, 5 Egr3-miR Cocaine,
4d volume ratio	Two-way ANOVA	Drug: $F_{(1,14)}$=3.008, P=0.1048, Virus: $F_{(1,14)}$=0.08972, P=0.7689, Interaction: $F_{(1,14)}$=3.919, P=0.0678.	4 SS-miR Saline , 4 SS-miR Cocaine 5 Egr3-miR Saline, 5 Egr3-miR Cocaine,
