# Supplementary material for: Cocaine-induced neuron subtype mitochondrial dynamics through Egr3 transcriptional regulation
Source: Mol Brain. 2021 Jun 29;14:101. doi: 10.1186/s13041-021-00800-y (PMC8240292; doi:10.1186/s13041-021-00800-y)
Supplement: Supplementary file 4 — Additional file 4. Gene regulation by species, brain region, and cell population. [file 13041_2021_800_MOESM4_ESM.pdf]

	Species	Nrf1	Nrf2	Poly	Tfam	Tfb1	Tomm20	Egr3	PGC1α	Drp1
**Total Brain**	human	-	↑	-	-	-	-	N/A	↑	↑[3]
	rat (self-admin)	↑	-	-	-	↑	↑	↓[1]	-	↑[3]
	mouse (i.p.)	↑	↑	-	-	-	↑	↓[1]	↑[2]	↑[3]
**D1 MSN**	mouse (i.p.)	-	-	-	↑	-	-	↑[1]	↑[2]	↑[3]
**D2 MSN**	mouse (i.p.)	-	-	↓	↓	↓	↓	↓[1]	↓[2]	↓[3]

[1]Chandra, R., et al., (2015). *Journal of Neuroscienc*e, 35(20), 7927–7937
[2]Chandra, R., et al., (2017). *Biological Psychiatry*, 81(7), 564-572
[3]Chandra, R., et al., (2017). Neuron*, 96(6), 1327-1341.e6*
